# Challenges in Delivering Academic Programmes: How Can We Achieve High Quality, Sustainable and Equitable Teaching Programmes Across Training Schemes?

**DOI:** 10.1192/bjo.2026.11294

**Published:** 2026-06-30

**Authors:** Kakali Pal, Meinou Simmons, Athif Ilyas

**Affiliations:** 1 Oxford Health NHS Foundation Trust, Oxford, United Kingdom; 2 Thames Valley Child & Adolescent Psychiatry Training Scheme, Oxford, United Kingdom; 3 Oxford University Hospitals NHS Foundation Trust, Oxford, United Kingdom

## Abstract

**Aims:**

The Thames Valley Child & Adolescent Psychiatry training scheme is one of the smallest UK schemes for the specialty. The previous academic programme consisted of small local group teaching. However, over time, it became increasingly difficult to fill the programme from a limited pool. Alongside this, with more virtual teaching affecting the teaching (positively and negatively), the feedback from residents indicated a clear need for action. Hence a decision was taken to join with three London programmes that were successfully running a combined academic programme. Considering the potential impact of merging with a large group conducted virtually, we identified a need to start with a one-year pilot, and monitor the quality, accessibility and learning impact of this.

**Methods:**

We consulted with the residents on joining the virtual programme, and started collating feedback after every session with an online form. The feedback was reviewed at the end of every term and discussed with resident representatives present. We also obtained feedback from our resident group on the quality of the academic teaching at the end of the pilot to support our decision on whether to continue.

**Results:**

Sessional feedback has helped gauge quality of speakers and shape future sessions. The overall feedback at the end of the pilot showed an overwhelmingly positive response.75% of the cohort responded, with everyone rating the main speaker sessions as good or excellent. Resident-led sessions were also rated positively, although comments reflected the difficulty of engaging in discussions virtually when presenting cases or journal clubs. The advantages of joining programmes included the content quality, breadth of topics, and convenience. Disadvantages included less face-face contact and reduced interactivity. Suggestions to mitigate included more in-person sessions, and access to recordings. Most people value small group teaching as a learning approach and also highly rated an in-person day in Oxford.

**Conclusion:**

We conclude overall that joining of academic programmes has been a success for our scheme. We have also been able to positively contribute to the larger scheme by bringing in a structured feedback process to the programme. However, we also highlight some ongoing challenges in delivering academic programmes moving forwards. Important questions remain about future development of academic programmes: will more schemes need to merge to sustain their viability if they face similar challenges – should there be a national programme for each specialty? What are the risks of this, and how can we maintain the important identity of smaller schemes?

